# A16 ROLE OF HIPPO SIGNALING EFFECTORS YAP&TAZ IN GASTRIC METAPLASIA AND TUMORIGENESIS

**DOI:** 10.1093/jcag/gwae059.016

**Published:** 2025-02-10

**Authors:** Z Ju, J sung, M Farag, P Fiset, L Ferri, A Gregorieff

**Affiliations:** Pathology, McGill University, Montreal, QC, Canada; Pathology, McGill University, Montreal, QC, Canada; Pathology, McGill University, Montreal, QC, Canada; Pathology, McGill University, Montreal, QC, Canada; Pathology, McGill University, Montreal, QC, Canada; Pathology, McGill University, Montreal, QC, Canada

## Abstract

NOT PUBLISHED AT AUTHOR’S REQUEST

Please acknowledge all funding agencies listed below

**Funding Agencies**: CIHRstudent is supported by CIHR CGS D and TRIANGLE Canada

